# Evaluation of acute kidney injury with pediatric-modified RIFLE criteria after pediatric cardiac surgery

**DOI:** 10.1186/cc9520

**Published:** 2011-03-11

**Authors:** P Zeyneloglu, A Pirat, E Baskin, A Camkiran, C Araz, M Ozkan, N Bayraktar, G Arslan

**Affiliations:** 1 Baskent University Hospital, Ankara, Turkey

## Introduction

Acute kidney injury (AKI) is a serious complication associated with increased morbidity and mortality in pediatric patients undergoing surgery for congenital heart disease. The aim of this study was to evaluate children with AKI after pediatric cardiac surgery using pediatric-modified RIFLE (pRIFLE) criteria and to investigate the value of serum cystatin C in patients with AKI.

## Methods

Eighty-one children undergoing cardiopulmonary bypass (CPB) for surgical correction of acyanotic congenital heart disease were prospectively enrolled in the study. Serial blood samples were collected to measure serum cystatin C and creatinine levels. The primary outcome measure was AKI, defined as ≥50% increase in serum creatinine from baseline.

## Results

Twenty-one children (26%) developed AKI, in which risk occurred in 12 (15%), injury in three (4%) and failure in six (7%) of the patients diagnosed with serum creatinine. Patients with AKI were significantly younger than patients without AKI (*P *= 0.002). No differences were noted with respect to CPB and aortic cross-clamp durations in those with and without AKI (*P *> 0.05). Postoperative 24-hour inotrope scores were significantly higher in children who developed AKI (*P *= 0.003). Serum cystatin C concentrations were significantly increased in AKI patients at 2 hours after CPB (*P *= 0.029) and remained elevated at 24 hours (*P *< 0.001) and 48 hours (*P *= 0.001). There was a significant positive correlation between presence of AKI and serum cystatin C levels (*P *< 0.05). A significant negative correlation was found between age and AKI (*r *= -0.344, *P *= 0.002).

## Conclusions

AKI develops in 26% of patients after pediatric cardiac surgery. Our results suggest that patients with AKI were younger and had postoperative higher serum cystatin C levels and higher inotrope scores when compared with patients without AKI.

## References

[B1] Krawchzeski CD Clin J Am Soc Nephrol 2010 5 1552 1557 10.2215/CJN.02040310 PMC2974393 20538834

